# Dietary Derived Propionate Regulates Pathogenic Fibroblast Function and Ameliorates Experimental Arthritis and Inflammatory Tissue Priming

**DOI:** 10.3390/nu13051643

**Published:** 2021-05-13

**Authors:** Jasna Friščić, Kerstin Dürholz, Xi Chen, Cecilia Engdahl, Lisa Möller, Georg Schett, Mario M. Zaiss, Markus H. Hoffmann

**Affiliations:** 1 Department of Internal Medicine 3—Rheumatology and Immunology, Friedrich-Alexander Universität Erlangen-Nürnberg (FAU), Universitätsklinikum Erlangen, 91054 Erlangen, Germany; jasna.friscic@uk-erlangen.de (J.F.); kerstin.duerholz@uk-erlangen.de (K.D.); xi.chen@extern.uk-erlangen.de (X.C.); lisamoeller2@web.de (L.M.); georg.schett@uk-erlangen.de (G.S.); mario.zaiss@uk-erlangen.de (M.M.Z.); 2 Deutsches Zentrum fuer Immuntherapie, Friedrich-Alexander Universität Erlangen-Nürnberg (FAU), Universitätsklinikum Erlangen, 91054 Erlangen, Germany; 3 Department of Rheumatology and Inflammation Research, Centre for Bone and Arthritis Research, Institute of Medicine, Sahlgrenska Academy, University of Gothenburg, 405 30 Gothenburg, Sweden; cecilia.engdahl@gu.se; 4 Department of Internal Medicine and Clinical Nutrition, Centre for Bone and Arthritis Research, Institute of Medicine, Sahlgrenska Academy, University of Gothenburg, 405 30 Gothenburg, Sweden

**Keywords:** arthritis, inflammatory tissue priming, propionate, diet, synovial fibroblasts, cellular senescence

## Abstract

Short-chain fatty acids are gut-bacteria-derived metabolites that execute important regulatory functions on adaptive immune responses, yet their influence on inflammation driven by innate immunity remains understudied. Here, we show that propionate treatment in drinking water or upon local application into the joint reduced experimental arthritis and lowered inflammatory tissue priming mediated by synovial fibroblasts. On a cellular level, incubation of synovial fibroblasts with propionate or a physiological mixture of short-chain fatty acids interfered with production of inflammatory mediators and migration and induced immune-regulatory fibroblast senescence. Our study suggests that propionate mediates its alleviating effect on arthritis by direct abrogation of local arthritogenic fibroblast function.

## 1. Introduction

The gut harbors trillions of bacteria that are constantly interacting with the host, thus representing a fundamental component underlying health and disease. Microbes often communicate with the host through microbiota-derived metabolites. One major class of metabolites are short-chain fatty acids (SCFAs). The SCFAs acetate, propionate (propionic acid, PA), and butyrate are the main metabolites produced in the gut by bacterial fermentation of dietary fiber and resistant starch. Besides local effects in the gut, SCFAs have several immunomodulatory capacities and exert effects on remote target organs.

For instance, PA was shown to shift Th1 and Th17 cells to a T regulatory cell (Treg) phenotype and thereby promote amelioration of multiple sclerosis [1] and allergic lung inflammation [2,3]. There is also a plethora of known effects that SCFAs have on myeloid cells. For example, SCFAs induce chemotaxis in neutrophils through free fatty acid receptor 2 [4,5] but also reduce nuclear factor (NF)-κB activation in these cells [6]. In monocytes, SCFAs modulate production of inflammatory mediators, such as cytokines, chemokines, and prostaglandins [7] and in macrophages, butyrate reduced activity of the mechanistic target of rapamycin (mTOR) [8]. We and others have shown that SCFAs are also able to attenuate local joint inflammation and associated bone destruction in the preclinical T-cell-dependent collagen-induced arthritis (CIA) model [9,10]. In CIA, SCFAs exhibited effects on the adaptive T-cell response, especially by inducing Tregs, and both butyrate and PA directly changed the metabolism of osteoclast (OC) progenitors, thus effectively suppressing their differentiation into mature bone degrading OC [9,10]. Accordingly, the gut microbiota is a strong regulator of systemic bone mass in mice, and SCFAs also regulated systemic bone mass in a noninflammatory model for postmenopausal bone loss [9,11]. At the same time, butyrate was shown to promote bone anabolism via Treg-mediated regulation of Wnt10b production by CD8^+^ T cells [12].

In addition to preclinical models, similar observations were reported following nutritional SCFAs supplementation in healthy humans [9] and individuals with rheumatoid arthritis (RA) [13,14]. In line with this, it is interesting to mention that several studies linked the composition of the gut microbiome to inflammatory bone loss in RA [15,16], spondyloarthritis, and ankylosing spondylitis [17,18,19].

However, in contrast to SCFAs’ modulation of the function of infiltrating immune cells [7], it is yet unresolved if SCFAs also act on joint resident cells that can harbor important impacts on arthritis pathogenicity in conjunction with or independently of the adaptive and innate immune systems [20,21]. We therefore studied whether systemic administration of PA and local PA injection into the joints affects T-cell-dependent and –independent murine arthritis models and found that PA ameliorates inflammatory arthritis by dampening pathogenic functions of synovial fibroblasts (SFs), such as migration, production of inflammatory mediators, and inflammatory tissue priming. Our data provide evidence that SCFAs can also tone down inflammation by direct action on tissue-resident stromal cells.

## 2. Materials and Methods

### 2.1. Mice

All mice belonging to BALB/c and C57BL/6 strains were bred at the University of Erlangen, Germany and reared under temperature- and humidity-regulated conditions. Mice were group housed with 12 h light/dark cycles and were given food and water ad libitum. Both male and female healthy adult mice that were 8–12 weeks old at the start of the experiment were used in the studies. All experiments included littermate controls with matched sex and age. Randomized assignment of mice into groups was performed by a random number generator (Randomizer. Available online: http://www.randomizer.org, accessed on 12 May 2021), so that both control and experimental group animals were represented in each cage to avoid eventual cage effects.

### 2.2. Experimental Arthritis Models

In order to elicit antigen-induced arthritis (AIA), C57BL/6 mice were subcutaneously immunized twice with 200 µg methylated bovine serum albumin (mBSA) emulsified in 100 µL Complete Freund’s adjuvant with 1 mg/mL *Mycobacterium tuberculosis*. Thereafter, a suspension of 100 µg mBSA was intra-articularly injected into the knee joint. Monosodium urate (MSU) crystal induced monoarthritis was instigated by injecting 1.4 mg MSU crystals suspended in 70 µL sterile PBS into the footpads of the hind paws, between the metatarsal bones. MSU crystals were synthetized in house, assuring the elimination of potential lipopolysaccharide (LPS) contamination [22]. Crystallization was performed by dissolving uric acid (10 mM) in 154 mM sodium chloride solution at pH 7.2. After the formation, crystals were rinsed with ethanol and decontaminated by dry heat sterilization at 180 °C for 2 h. Zymosan-induced arthritis was elicited by injecting 0.3 mg zymosan (Sigma Aldrich, Z4250, St. Louis, MO, USA) suspension in PBS. Firstly, zymosan was administered into the metatarsal area of the left hind paw only. After resolution of the first arthritis episode, zymosan was then injected into both hind paws.

To monitor the severity of arthritis models, paw swelling was measured with an electronic caliper (Kroeplin, Schluechtern, Germany). Obtained scores were normalized and presented as areas under the arthritis curves (AUC). Priming index was calculated as the ratio between the AUC of the second episode and the concurrent first arthritis episode in the contralateral paw.

### 2.3. Treatment of Animals with Propionic Acid (PA)

Mice were treated with 150 mM sodium propionate (Sigma Aldrich, P1880, St. Louis, MO, USA) in the drinking water as described [9]. Control mice received pH and sodium-matched water. For the local administration, 30 µL mBSA solution or MSU crystal suspension was coinjected with or without 1 mM propionate. Morphology of MSU crystals after incubation with sodium propionate was controlled by light microscopy and was not changed.

### 2.4. Inflammatory Tissue Priming Model by Cell Transfer

MSU crystal suspension was administered to the left hind paws of mice. After the inflammation had resolved, 6 × 10^5^ sorted SFs derived from primed paws that had been injected twice with MSU crystals as described [21] were transferred to the metatarsal area of the contralateral paw by subcutaneous injection. The control group received untreated SFs, while the treatment group received SFs cultured with 250 µM propionate for 24 h at 37 °C. Two days following cell transfer, arthritis was elicited in both paws by injecting MSU crystal suspension.

### 2.5. Histology

Histomorphological evaluation was performed on knee sections from both naïve mice and mice with antigen-induced arthritis. After the dissection, samples were fixed in 4% formalin. After the decalcification with EDTA, specimens were embedded in paraffin blocks. Sections were stained with H&E (haematoxylin and eosin) and TRAP (tartrate-resistant acid phosphatase), after which inflammatory changes and osteoclasts were analyzed on a Nikon microscope (Tokyo, Japan).

### 2.6. Isolation and Culturing of Synovial Fibroblasts from Mouse Paws

Primary synovial fibroblasts (SFs) were isolated from noninjected naïve paws or from primed paws injected twice with MSU crystals as described [21]. After the dissection, skin, tendons, and muscle were removed from paws. SFs cell suspensions were prepared by digesting the paws in 2 mg/mL collagenase type IV (Worthington Biochemicals, LS004188, Lakewood, CA, USA) solution in DMEM for 1 h at 37 °C. The cell suspension was then filtered, and the cells collected by centrifugation. In order to obtain higher yield, primary cells from 2 paws were combined and then kept in culture in Dulbecco’s modified eagle medium (Gibco, Carlsbad, CA, USA) containing 10% heat-inactivated fetal calf serum and 1% each of penicillin, streptomycin, and fungicide (Amphotericin B, Sigma Aldrich, St. Louis, MO, USA). After two passages, cultured cells were detached using Corning Cell stripper™ (Corning 25-056, Corning, NY, USA) and stained with Sytox Green (Thermofisher S7020, Waltham, USA), BV421-coupled anti-CD31, and APC-coupled anti-CD45 antibodies. Cells were sorted on a FACS Aria machine, and CD45^-^ CD31^-^ negative cells were recultured. SFs between passages 4 and 8 were used for the experiments.

### 2.7. Treatment of SFs with SCFAs In Vitro

Cultured SFs were treated for 24 h with 250 µM propionate or a physiological mixture of the SCFAs acetate, propionate, and butyrate (300 µM acetate, 100 µM propionate, 100 µM butyrate, all from Sigma-Aldrich, St. Louis, MO, USA). LPS stimulation of the SFs was achieved by adding 200 ng/mL LPS from *E. coli* to the growth medium for 24 h. In vitro treatment with SCFAs used concentrations that were previously shown to be nontoxic to cells [9].

### 2.8. Quantitative Real-Time Polymerase Chain Reaction

RNA from mouse SFs was extracted using the RNeasy kit (Qiagen 74104, Hilden, Germany) according to the manufacturer’s instructions. RNA quality and quantity was determined by spectrophotometric optical density measurement (NanoDrop, Wilmington, NC, USA). For cDNA synthesis, 1 µg of total RNA per sample was reverse transcribed. Gene expression profiles were determined by real-time quantitative PCR on the Bio-Rad CFX96 Touch Real-Time PCR Detection System (Hercules, CA, USA). SYBR Green fluorescent dye-based MasterMix (Eurogentec RT-SN2X-03, Leige, Belgium) was used for the amplification and detection. Gene expression values of target genes were normalized relative to the expression of the reference gene (*Actb*). Sequences of the primers used in the experiments are listed in the Table 1 below.

### 2.9. Scratch Assay

Two-well cell culture-inserts (IBIDI, 80209, Munich, Germany) were disinfected for 20 min in 70% ethanol. After having fully dried, the inserts were carefully inserted into a 12-well cell culture plate. 25,000 SFs from naïve mouse paws (passage 4) were seeded into each well of the inserts in medium ±250 µM PA (Sigma-Aldrich, P1880, St. Louis, MO, USA). Inserts were carefully removed after 12 h, and 1 mL of the respective medium (±250 µM PA) was added. Cell migration was monitored for 72 h in 15 min intervals by live cell imaging with an automated microscope (Zeiss CellDiscoverer 7, Jena, Germany).

### 2.10. Analysis of Fibroblast Senescence

Naïve murine SFs (10 × 10^3^) were stimulated for 24 h on 12-well cell culture plates by adding 10 ng/mL TNFα to the culture medium. After the stimulation and a thorough wash with PBS, they were left untreated in standard medium for 24 h, followed by a second stimulation with TNFα (10 ng/mL) for another 24 h. After the second TNFα stimulation, cells were washed with PBS and treated for 24 h with medium supplemented with 250 µM propionate. Control group was comprised of cells which were cultured without any stimulation with TNFα. Additionally, MSU-primed aged naïve synovial fibroblasts from passage 7 were assessed without TNF stimulation and with/without 24 h treatment with 250 µM propionate. SA-β-Gal activity was measured using a senescence detection kit (Abcam, 65351, Cambridge, UK).

### 2.11. Statistical Analysis

Paired or unpaired Student’s *t*-test was used for making two group comparisons. For non-normally distributed data, 2-tailed Mann–Whitney U test was applied. Normal Gaussian distribution of obtained data was estimated by D’Agostino–Pearson omnibus normality test. Multiple comparisons among groups in every set of experiments represented on a single graph were performed by ANOVA, followed by Sidak’s multiple comparisons test. Adjusted *p*-values lower than 0.05 were considered statistically significant. Data analysis and visualization was done using GraphPad Prism 8.3 software package (San Diego, CA, USA). Levels of significance are allocated as follows throughout the whole manuscript: * *p* < 0.05; ** *p* < 0.01; *** *p* < 0.001.

## 3. Results

### 3.1. Systemic or Local Administration of Propionic Acid Ameliorates Inflammatory Arthritis and Tissue Priming

**Figure 1:**
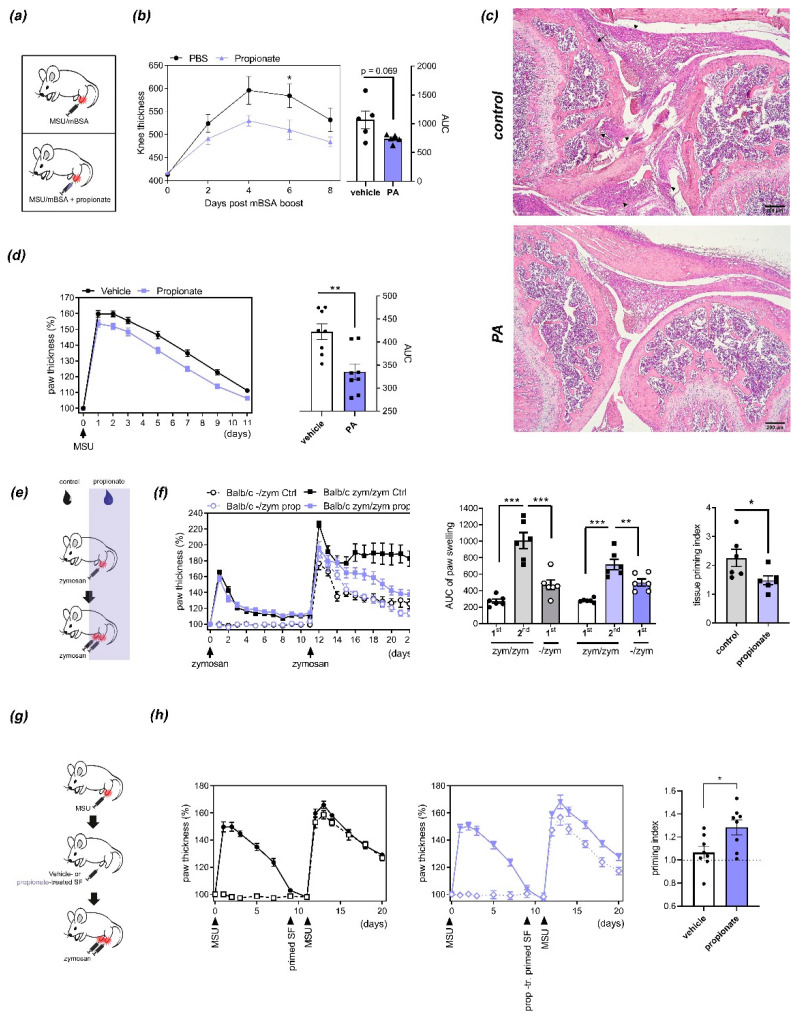
Effects of propionic acid (PA) on arthritis and inflammatory tissue priming: (**a**) Injection schemes for antigen-induced arthritis (AIA) and MSU crystal-induced arthritis. (**b**) Time course (left) and area under the curve (AUC, right) of relative thickness of C57BL/6 mouse paws (n = 5) injected with methylated bovine serum albumin (mBSA) with or without PA. * *p* < 0.05, ANOVA with Sidak´s multiple comparisons test. *p* = 0.069, Student´s *t*-test. (**c**) Inflammatory and bone changes in AIA induced by injection of mBSA with or without PA. Representative H&E staining of sections from mouse paws 8 days after mBSA boost. Black arrowheads indicate inflammatory infiltrates, black arrows areas of bone/cartilage destruction. Scale bars, 200 µm. (**d**) Time course (left) and AUC (right) of arthritis induced by injection of MSU crystals with/without PA. N = 8. ** *p* < 0.01, Student´s *t*-test. (**e**) Injection scheme for the inflammatory tissue priming model of arthritis upon treatment with PA in the drinking water. (**f**) Time course of paw thickness (left), AUCs (center) and priming indices (right) during iterated zymosan-induced arthritis in BALB/c mice treated with or without PA in the drinking water (n = 6). Priming index represents the ratio between AUC of the second episode of arthritis and the first episode of arthritis in the contralateral paw. * *p* < 0.01, ** *p* < 0.01, *** *p* < 0.001, ANOVA with Sidak´s multiple comparisons test (AUC); Student´s *t*-test (priming index). (**g**) Injection scheme of inflammatory tissue priming by transfer of SFs. (**h**) Course of arthritis (left and center) and priming indices (right) after transfer of primed PA- or vehicle-treated SFs into the naïve paws of recipient mice (n = 8). * *p* < 0.05, Student´s *t*-test.

The systemic administration of SCFAs was previously shown to reduce autoimmune disease by modulating T-cell-dependent autoimmunity [1,9,10]. To assess if propionate is also functional when administered locally, we used antigen-induced arthritis (AIA), a T-cell-dependent model of arthritis that relies on systemic immunization with methylated bovine serum albumin (mBSA) preceding local initiation of inflammation by intra-articular knee injection of mBSA [23]. When coinjected intra-articularly with mBSA at physiologically relevant concentrations [9], PA significantly reduced knee swelling at day 6 after injection (Figure 1a,b) and ameliorated histological signs of inflammation (Figure 1c). To determine if this effect of PA relies on local modulation of T-cell function or potentially also other cell types, we assessed its effect on an innate immunity-driven arthritis model. To this end, mice were injected with zymosan, a yeast-derived damage-associated molecular pattern (DAMP), or MSU crystals, which both trigger destructive arthritis independently of adaptive immunity [21]. Similarly to AIA, coinjection of PA together with MSU crystals reduced paw swelling (Figure 1d). Interestingly however, administering PA systemically via the drinking water as previously described [9] had no significant effect on acute innate immunity-driven arthritis but reduced the worsening of arthritis after repeated injection of the inflammatory trigger (inflammatory tissue priming) (Figure 1e,f). Inflammatory tissue priming is driven by local priming of resident SFs [21]. To assess the effects of PA on the arthritogenicity of fibroblasts, we treated primed SFs (derived from paws that had been injected twice with MSU crystals) with nontoxic concentrations of PA and transferred them in an MSU crystal-driven model as described [9,21] (Figure 1g). PA-treated cells were significantly impaired in their ability to transfer tissue priming to naïve paws (Figure 1h), suggesting that PA directly impairs SF function related to arthritis and inflammatory tissue priming.

### 3.2. Propionate Treatment Interferes with Arthritogenic Properties of SFs and Induces Cellular Senescence

**Figure 2:**
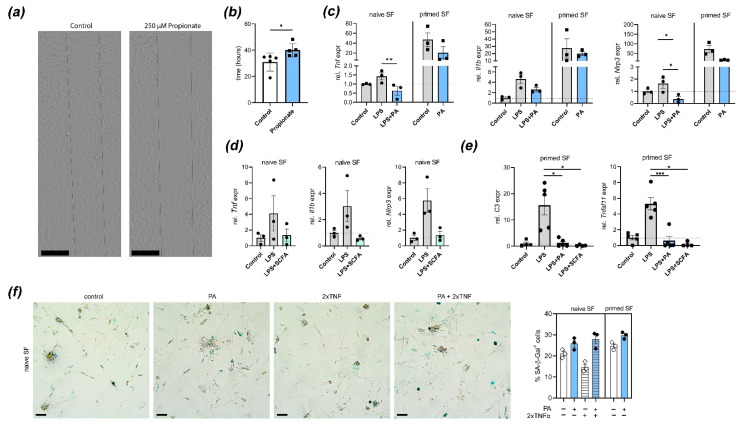
Treatment with propionic acid (PA) or SCFA blocks arthritogenic fibroblast function: (**a**,**b**) Fibroblast migration is reduced by treatment with PA. Representative images (**a**) of a wound healing/migration assay with murine SFs isolated from C57BL/6 mice. Dashed lines show cell-free space at experiment start. Scale bars, 400 µm (**b**) Quantification of wound healing/migration (time until confluency) in PA-treated and control murine SFs (n = 5). * *p* < 0.05, Student´s *t*-test. (**c**) Quantitative real-time PCR analysis in cultured naïve and primed mouse SFs stimulated with LPS with or without PA. Shown are expression of *Tnf*, *Il1b* and *Nlrp3*. * *p* < 0.05, ** *p* < 0.01, ANOVA with Sidak´s multiple comparisons test. (**d**) qPCR analysis in cultured naïve SFs stimulated with LPS with/without physiological mixture of short-chain fatty acids (SCFAs; acetate, propionate, butyrate). Plotted are individual values and means of *Tnf*, *Il1b* and *Nlrp3* expression. (**e**) qPCR analysis of LPS-stimulated and control SFs treated with PA or SCFA mixture. Shown is expression of *C3* and *Tnfsf11* (RANKL). * *p* < 0.05, *** *p* < 0.001, ANOVA with Sidak´s multiple comparisons test. (**f**) Representative images and quantification of propionate or vehicle-treated murine synovial fibroblast stained for senescence-associated β-galactosidase (blue). Scale bars, 200 µm. All subfigures show individual values, means and S.E.M. Each symbol represents pooled cells from 2 mice.

In paws pre-exposed to an inflammatory attack, joint-resident SFs undergo adaptation processes that prime subsequent local responses. These “primed” SFs are characterized by an aggressive SF phenotype including increased migration, production of proinflammatory mediators, and NLRP3 inflammasome activation [21]. To determine how treatment with PA impacts SF function, we first performed a scratch assay in murine in vitro PA-treated and control SFs. Treatment with PA reduced wound healing/migration in this assay (Figure 2a,b). PA treatment also inhibited LPS-induced expression of *Tnf*, *Il1b* and *Nlrp3* in naïve SFs and reduced spontaneous expression of these genes in primed SFs derived from mouse paws that had been injected twice with MSU crystals (Figure 2c). A mixture of SCFAs in the physiologically relevant ratio of acetate:propionate:butyrate = 3:1:1 (equalling 300 µM acetate, 100 µM propionate, and 100 µM butyrate) also inhibited LPS-induced upregulation of inflammatory mediator expression (Figure 2d). Pathogenic fibroblast function in arthritis and tissue priming is associated with complement-mediated metabolic reprogramming and bone destruction [21]. To determine if PA alone or a physiological SCFA mixture potentially intervene with these processes, we performed expression analysis of *C3* and *Tnfsf11* (RANKL). Treatment with either PA alone or SCFA mixture reduced *C3* and *Tnfsf11* expression upon stimulation with LPS in primed SFs (Figure 2e), suggesting that the effect of SCFAs might abrogate metabolic invigoration and tissue destuction associated with tissue priming. Furthermore, PA treatment induced cellular senescence in SFs, which was previously shown to be associated with regulation and resolution of inflammation [21,24].

## 4. Discussion

While fibroblasts have classically been considered merely structural cells whose primary function is building and restructuring the extracellular matrix, it has become clear that they play a multifaceted role in health and disease. In particular, fibroblasts have emerged as important immune-sentinel cells that modulate immune responses upon the detection of pathogen- or damage-associated molecular patterns (PAMPs and DAMPs, respectively), aid in recruitment of leukocytes, and shape inflammation. Therefore, fibroblasts are now recognized as a non-classical part of the innate immune system [25].

The resident tissue of inflamed joints is characterized by a tight network of SFs interacting with resident macrophages, endothelial cells, and infiltrating myeloid cells. During arthritis, SFs gradually acquire a more disease-promoting phenotype. This aggressive state includes mounting augmented production of proinflammatory mediators, a proliferative and invasive cell behavior triggering tissue destruction, and the promotion of leukocyte trafficking into joints [26,27,28,29,30,31]. Modern cellular phenotyping has uncovered that a Thy1^+^CD34^+^ subset of SFs, which expresses several complement proteins intracellularly and is found in the sublining regions of the synovium, critically contributes to arthritis pathogenicity [32,33,34,35]. We have recently shown that this SF subset is also responsible for inflammatory tissue priming, i.e., a prolonged response to repeated inflammatory triggers, while resident macrophages play a negligible role in this process [21]. Inflammatory tissue priming provides an explanation for relapses of inflammation in the same tissue and for the transition from self-limited to chronic inflammation, which is a hallmark of inflammatory arthritides such as RA or gout.

Apart from the role in shaping sterile inflammation, tissue-resident fibroblasts exert physiological functions in direct response to microbial signals. For example, in intestinal chronic inflammation and fibrosis, specific microbiota are required for fibroblast activation and transformation into myofibroblasts [36]. Under germ-free conditions, reduced fibroblast migration and activation was observed. Additional evidence is now beginning to emerge that microbial metabolites are also directly sensed by fibroblasts and modulate inflammation and fibrosis in mice [37]. Direct effects of SCFAs on structural cells are mostly known from fibroblasts of the periodontal pocket that are in direct contact with the microbiota [38,39] and skin fibroblasts [40]. Given the apparent impact of SCFAs such as PA on sterile chronic inflammation in the joint, the potential impact on joint-resident cells merited further investigation.

Here, we provide evidence that PA, beside its described systemic effects on adaptive immune cells, modulates the functional phenotype of arthritogenic SFs. In order to best dissect local from systemic responses and investigate potential spatiotemporal effector mechanisms of PA, we applied PA as a single injection directly into the knee joint or paw, transferred primed and ex vivo PA-treated SFs into recipient paws, or additionally assessed local SF-following systemic PA treatment. In all three experimental designs, PA attenuated inflammatory arthritis and supporting in vitro findings let us conclude that the direct anti-inflammatory effects of local fibroblasts in part mediate these positive clinical outcomes. In particular, treatment with PA at concentrations occurring under dietary supplementation also in vivo [9] or a physiological mixture of the SCFAs acetate, propionate, and butyrate inhibited LPS-induced induction of inflammatory mediators that have a relevance in arthritis and reduced their expression in MSU-primed fibroblasts [41,42]. PA also arrested SF migration. Most importantly, treatment of primed SFs with PA inhibited their priming, as shown from their reduced capacity to induce a primed state in previously non-injected paws [21]. Mechanistically, our data suggest that PA reduces arthritogenic fibroblast function by interfering with complement-mediated metabolic activation of SFs and thus by channeling them towards a senescent, regulatory phenotype upon activation with inflammatory stimulants [21,24].

Our study was first set to generate comprehensive insights into the dietary-derived SCFA production in the gut and the local cellular consequences for SF activation during inflammatory arthritis. We unraveled PA-mediated effects in SFs on checkpoints of immune tolerance and tissue protection. Further analysis will be needed to completely dissect the underlying molecular mechanisms. In summary, we provide additional arguments that diet emerges as a pivotal determinant of the microbial metabolites as immunologic effector molecules. By introducing dietary signals into the nexus between the microbiota and the host immune system, nutrition can sustain immune homeostasis and contribute to the prevention of inflammatory diseases such as RA.

## Figures and Tables

**Table 1:** Primers used for the study.

Gene	Forward (5′-3′)	Reverse (5′-3′)
*Actb*	TGTCCACCTTCCAGCAGATGT	AGCTCAGTAACAGTCCGCCTAGA
*Il1b*	AACCTGCTGGTGTGTGACGTTC	CAGCACGAGGCTTTTTTGTTGT
*Tnf*	CAGGCGGTGCCTATGTCTC	CGATCACCCCGAAGTTCAGTAG
*Nlrp3*	TGCTCTTCACTGCTATCAAGCCCT	ACAAGCCTTTGCTCCAGACCCTAT
*C3*	GACGCCACTATGTCCATCCT	CCAGCAGTTCCAGGTCCTTTG
*Tnfsf11*	TGTACTTTCGAGCGCAGATG	AGGCTTGTTTCATCCTCCTG

## Data Availability

The data presented in this study are available on request from the corresponding author.
